# Smart Surface of Steering Wheel Based on Triboelectric Nanogenerator and Artificial Intelligence in Driving Monitoring System

**DOI:** 10.3390/nano15191472

**Published:** 2025-09-25

**Authors:** Fengguang Fan, Shiju E, Mengzhe Kang, Yuting Fan, Qing Song, Yu Xie, Yuankai Zhou, Jiaheng Nie, Xin Cui, Yan Zhang

**Affiliations:** 1 College of Engineering, Zhejiang Normal University, Jinhua 321004, China; fanfengguang@zjnu.edu.cn; 2 Beijing Key Laboratory of High-Entropy Energy Materials and Devices, Beijing Institute of Nanoenergy and Nanosystems, Chinese Academy of Sciences, Beijing 101400, China; mengzhe.kang@std.uestc.edu.cn (M.K.); yutingfan@std.uestc.edu.cn (Y.F.); songqing25@mails.ucas.ac.cn (Q.S.); xyu2024@lzu.edu.cn (Y.X.); 3 School of Nanoscience and Engineering, University of Chinese Academy of Sciences, Beijing 100049, China; 4 School of Physics, University of Electronic Science and Technology of China, Chengdu 610054, China; yuankai_zhou@cdtu.edu.cn; 5 School of Electronic Engineering, Chengdu Technological University, Chengdu 611730, China; 6 School of Cybersecurity (Xin Gu Industrial College), Chengdu University of Information Technology, Chengdu 610225, China; njh@cuit.edu.cn; 7 School of Materials and Environmental Engineering, Chengdu Technological University, Chengdu 611730, China; cuixin@cdtu.edu.cn

**Keywords:** triboelectric nanogenerator, smart surface, driving monitoring, machine learning

## Abstract

Real-time driving monitoring systems can use self-powered sensors based on artificial intelligence (AI) and triboelectric nanogenerators (TENGs). Here, we created a TENG-based self-powered intelligent steering wheel that can detect hand gripping. The TENG serves as the steering wheel’s smart surface. In addition to monitoring the steering wheel in real time, the intelligent steering wheel reacts quickly. The TENG sensor can detect hazardous conditions and lower processing demands while retaining excellent identification accuracy when used in conjunction with machine learning. Additionally, the TENG sensor may now offer an accurate and affordable monitoring solution for smart driving thanks to the integration of AI.

## 1. Introduction

Triboelectric nanogenerators (TENGs) are an innovative technology for energy harvesting and have attracted significant global attention since their inception [1,2]. TENG sensors are widely utilized for respiration monitoring, vibration and sound sensing, tactile and pressure measurements, and wearable sensor technologies [3,4,5,6]. A number of TENG sensors that can track and recognize a wide range of human activities, such as hand motions, breathing, posture, walking patterns, lip movements, and waist activity, have been developed recently [7,8,9,10,11,12]. Previous advances focused on the application of TENG-based human–computer interface sensors for identifying driver behaviors in semi-automated driving scenarios remain scarce. The introduction of highly automated driving systems in automobiles is considered a revolutionary strategy to increase the effectiveness of urban transportation on a broad scale [13]. In order to maintain operational safety, Level 3 autonomous driving systems require drivers to take on crucial vehicle takeover duties [14]. If they need to take control again, drivers must continue to hold their hands on the steering wheel and concentrate on the road ahead. The drivers are exposing themselves to serious danger when they take their hands off the steering wheel to do things like take a nap or answer the phone [15,16].

Automotive sensors were utilized to promote safe driving, including driver fatigue and early health monitoring and driving ability-related stress and emotional state detection [17]. For example, the biosensor can acquire electrophysiological markers of inattentiveness using electrodes integrated on the surface of steering wheels, but maintaining constant skin contact is challenging [18]. Camera-based monitoring techniques rely on indirect behavioral cues rather than physiological markers, are dependent on ambient lighting, and may cause privacy issues. Contactless sensors can help alleviate some of these issues to protect user privacy and are less susceptible to environmental noise, such as radar, Wi-Fi and photonic radar sensing systems [19,20,21]. Seat-embedded antennas [22], phased-array radar systems [23], and Radio Frequency Identification (RFID) [17] have also been investigated for this purpose. However, motion artifacts and signal interference from multipath reflection within the enclosed cabin make it difficult to obtain high-quality physiological parameters in dynamic environments with vibrations, random body movements, and multiple passengers.

Autonomous driving currently faces numerous challenges, not least of which is the driver’s hands-on control of the steering wheel. Currently, both the autonomous driving and even automotive industries are eagerly awaiting the application of TENGs. Autonomous driving technologies demand a prototype that combines the low-cost, simple TENG with a camera-based AI system while significantly reducing computing power and energy consumption requirements.

In this work, TENG sensors with artificial intelligence were developed to identify driver behaviors and provide timely alerts. The TENG sensor circuit was used to transmit information to an optical image sensing system. TENG and machine learning techniques are combined to create a TENG-based smart surface sensing system, which can be a high-performance, real-time sensor system for an autonomous driving scenario. This self-powered device can successfully warn of the potentially dangerous situation when the driver’s hands are not on the steering wheel. This method reduces the computational load on the car’s onboard systems and frees up computational resources for other vital safety features. The TENG-based smart surface paves a new way to develop a smart driving system based on triboelectric nanogenerator sensors and artificial intelligence.

## 2. Result and Discussion

**Figure 1:**
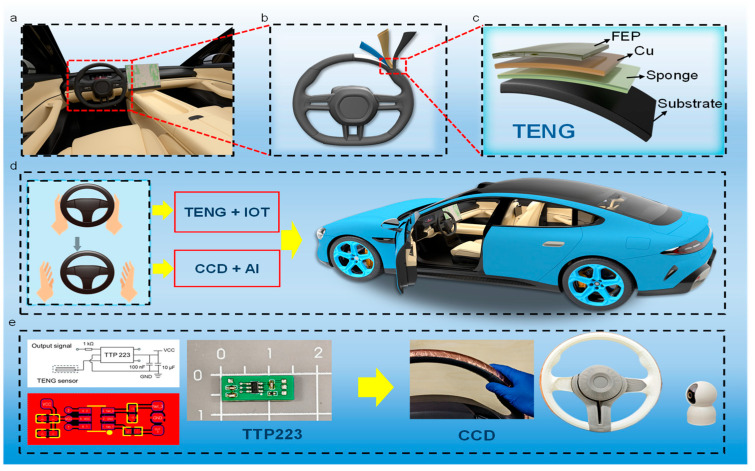
Schematic of smart surface in smart driving safety monitoring system. (**a**,**b**) The TENG-based smart surface is attached to the steering wheel. (**c**) The surface is composed of four layers: FEP film, Cu electrode, sponge and substrate. (**d**) When the driver’s hand leaves the steering wheel, the monitoring system will be triggered by the TENG sensor and IoT and invoke the CCD and AI to recognize the driver’s hand interaction status and give response to the car. (**e**) The schematic and layout of the custom-fabricated printed circuit board of the detection circuit, and the photograph of steering wheel holding detection monitoring systems.

The TENG smart surface integrates a triboelectric sensor capable of real-time detection of hand interaction with the steering wheel. A smart driving safety monitoring system is illustrated in Figure 1. There are three components: a TENG-based smart surface, an optical driver behavior capture module, and an AI recognition module. When the driver’s hand contacts or leaves the steering wheel, the smart surface generates a series of electrical signals. These signals, processed by a pulse detection circuit, activate the optical image capture module and the AI recognition module to assess the driver’s hand interaction status.

Based on the recognition results and AI judgment module, the system determines the driver’s behavior in real time. It can issue warning signals in response to unsafe driving behaviors to ensure vehicle safety. For instance, when the driver’s hand leaves the steering wheel, the integrated triboelectric sensor detects the event, generating a series of electrical pulses that trigger the optical image acquisition module.

The optical image capture module, equipped with a CCD sensor and a microprocessing unit, promptly captures the driver’s hand movements and transmits this information to the AI recognition module. The AI module, employing a deep learning model, analyzes whether the driver is properly holding the steering wheel. This system can assist with real-time correction of driving behavior and is compatible with existing Level 0–3 autonomous driving systems, providing effective support to drivers and enhancing driving safety.

### 2.1. Design and Fabrication of Single-Electrode Flexible TENG

Without sacrificing comfort or driving safety, flexible single-electrode triboelectric nanogenerators (TENGs) were incorporated onto the steering wheel surface (Figure 1a,b). The resulting lightweight, flexible, and highly sensitive technology allows for real-time hand position monitoring and lowers the risk of driver distraction.

A fluorinated ethylene propylene (FEP) triboelectric layer, a copper (Cu) electrode, an elastic sponge interlayer, and a supporting substrate make up the four functional layers that make up the TENGs, as shown in Figure 1c. In particular, the substrate provides mechanical support, the sponge interlayer provides flexibility and compressibility, the Cu layer acts as the signal collection electrode, and the FEP film acts as the principal triboelectric layer. This special design improves the device’s electrical output performance while also guaranteeing improved flexibility, mechanical stability, and wear resistance.

The proposed smart monitoring system leverages the structural advantages of the TENG-based smart surface in combination with advanced sensing and analysis technologies, as shown in Figure 1d. Specifically, the TENG sensor instantly recognizes the change in contact state when the driver’s hand leaves the steering wheel and sends this data to the monitoring system through the Internet of Things (IoT). The monitoring system then uses artificial intelligence (AI) algorithms and a CCD-based vision module to identify and interpret the driver’s hand movements.

### 2.2. Energy Harvesting Performance of TENGs

**Figure 2:**
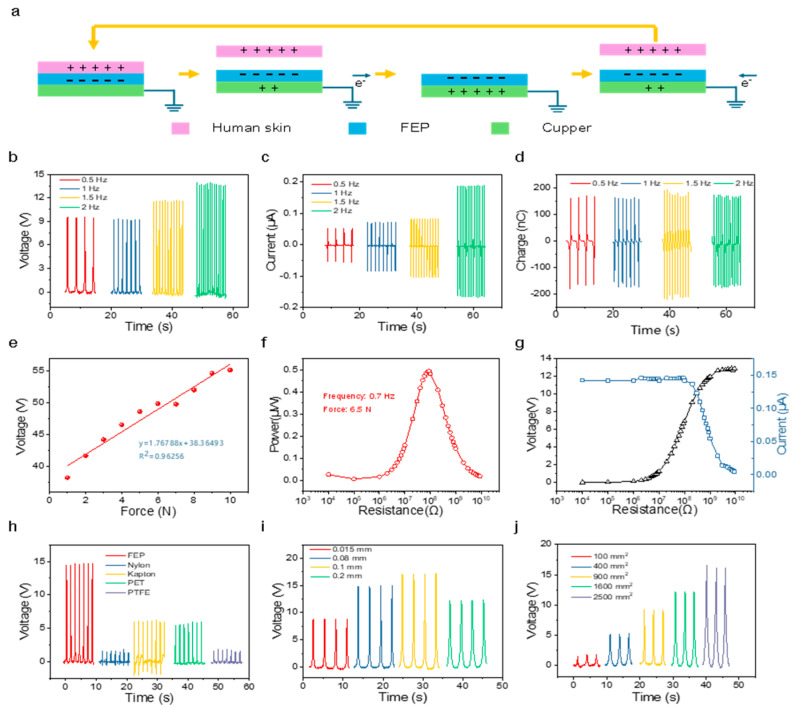
The output characteristics of the TENG device. (**a**) TENG working mechanism, (**b**) open−circuit voltage, (**c**) short−circuit current, and (**d**) transferred charge under different operating frequencies. (**e**) The changes in output voltage under different applied pressures. Relationships between (**f**) load voltage, current, and (**g**) output power under different load resistances. Figure 2g shows a comparison of the open−circuit voltage outputs for (**h**) various triboelectric materials, (**i**) device area and (**j**) triboelectric materials with different thicknesses.

As illustrated in Figure 2, the electrical response of the TENG under various external conditions and structural parameters was methodically examined in order to fully assess its output performance. The open-circuit voltage, short-circuit current, and transferred charge were measured using a Keithley 6514 electrometer. A National Instruments USB-6346 multifunction I/O device was employed for data acquisition. For load impedance measurements, a ZX21g rotary DC resistance box was utilized. Figure 1a shows the working mechanism of the TENG. Firstly, tests were conducted on the open circuit voltage, short circuit current, and transferred charge of TENG at various excitation frequencies (0.5 Hz, 1 Hz, 1.5 Hz, and 2 Hz) (Figure 2b–d). The results show that the output signal amplitude of TENG increases significantly with higher excitation frequencies, demonstrating excellent frequency response characteristics. This further validates the potential of TENG for use in dynamic sensing applications.

The investigation on the connection between output voltage and external force is shown in Figure 2d. According to the experimental data, there is a good linear relationship between the increase in external force and the output voltage of TENG. The device has high linearity and sensitivity in force sensing, as evidenced by the fitting line’s correlation coefficient R^2^ of 0.96256 and sensitivity of 1.77 V/N.

By varying the external resistance, the output power, open circuit voltage, and short circuit current of TENG are measured in relation to load characteristics (Figure 2e,f). The results indicate that the output power first increases and then decreases with increasing load resistance, reaching a peak of 0.5 μW at approximately 100 MΩ. In line with the output characteristics of a conventional TENG, the output voltage rises as resistance increases while the short-circuit current progressively falls. In Figure 2e,f, the actuation frequency was set to 0.7 Hz. A linear motor was employed to apply a normal pressure onto the TENG, operating in a two-point motion mode with a distance of 60 mm, where the motor drove the TENG through uniform acceleration and deceleration of 0.48 m/s^2^. The maximum applied pressure during this process was 6.5 N. The characterization and electrical measurement instrument are shown in Figure S2.

A range of common materials, including FEP, Nylon, Kapton, PET, and PTFE, was chosen for comparative experiments in order to investigate the impact of friction materials on TENG performance (Figure 2g). The results indicate that different friction materials have a substantial effect on the output voltage, with FEP providing the best performance. Furthermore, a thorough investigation was conducted into the regulatory impact of the effective contact area (Figure 2i) and the friction layer thickness (Figure 2h) on the TENG output. The output voltage exhibits a steady upward trend as the thickness of the friction layer and contact area increase, further extending the controllability and useful application range of TENG devices. The photographs of the front and cross-sectional views of the actual device are shown in Figure S1.

### 2.3. Wireless Signal Transmission and Machine-Learning Decoding

**Figure 3:**
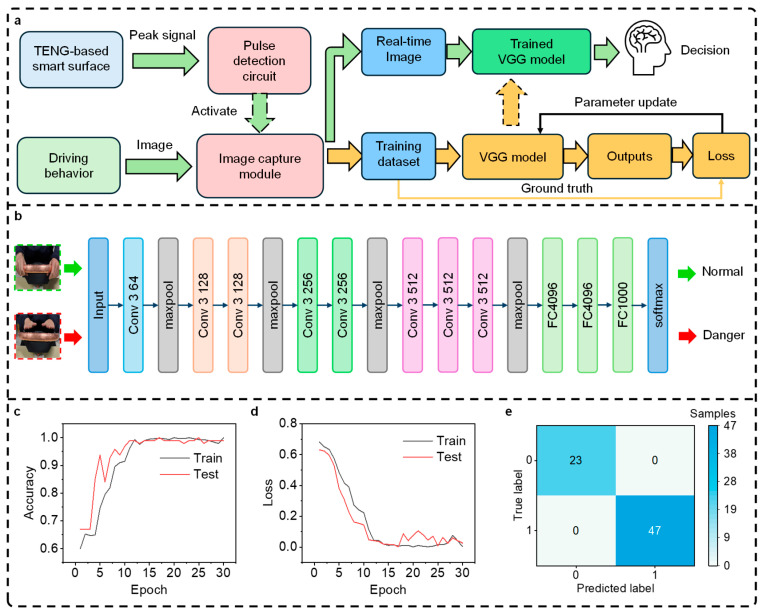
The working principle of the smart surface sensing system based on machine learning. (**a**) The data processing workflow for the deep learning-based steering wheel hand detection model during both the training and testing phases. (**b**) The architecture of the VGG model. (**c**) The prediction accuracy and (**d**) the loss function for the training and testing sets. (**e**) The confusion matrix of the prediction results.

Figure 3 shows the working principle of the smart surface sensing system based on machine learning. When the signal generated by the TENG-based smart surface is received by the pulse detection circuit, the optical image capture module is activated.

Figure 3a depicts the data processing workflow for the deep learning-based steering wheel hand detection model during both the training and testing phases. In the training phase, images of driving behavior captured by the image capture module are labeled and used to train the Visual Geometry Group (VGG) model. The error between the outputs predicted by the VGG model and the ground-truth labels was quantified using a loss function, and model parameters were subsequently updated by stochastic gradient descent. The initial learning rate for SGD was fixed at 0.0001. The VGG model was trained for 30 epochs in total. Finally, the trained VGG model is utilized to evaluate the safety of driving behaviors from real-time image data.

The architecture of the VGG model is shown in Figure 3b. The VGG model consists of eight convolutional and rectified linear unit (ReLU) layers, five pooling layers, and three fully connected layers. The real-time images are in color, with a standardized resolution of 640 × 640 × 3. In Figure 3e, the test set included 70 samples in total (23 + 47). The complete dataset comprised 350 samples, which were divided into training and testing sets. Specifically, we employed an 80–20% split, i.e., 280 samples for training and 70 samples for testing. This ratio is widely adopted in machine learning model evaluations as a standard practice to ensure sufficient training data while retaining a robust test set for performance assessment [24].

Figure 3c presents the training accuracy and testing accuracy of the VGG model. Figure 3d depicts the corresponding training and test errors of the model during the training process. The gradually converging loss curve suggests that the training process is stable. After 30 epochs of training, the VGG model achieved a test accuracy of 100%, with a corresponding test error of 0.03. Figure 3e shows the confusion matrix for the identification results.

### 2.4. Smart Surface of Steering Wheel Based on TENG and AI Sensors

**Figure 4:**
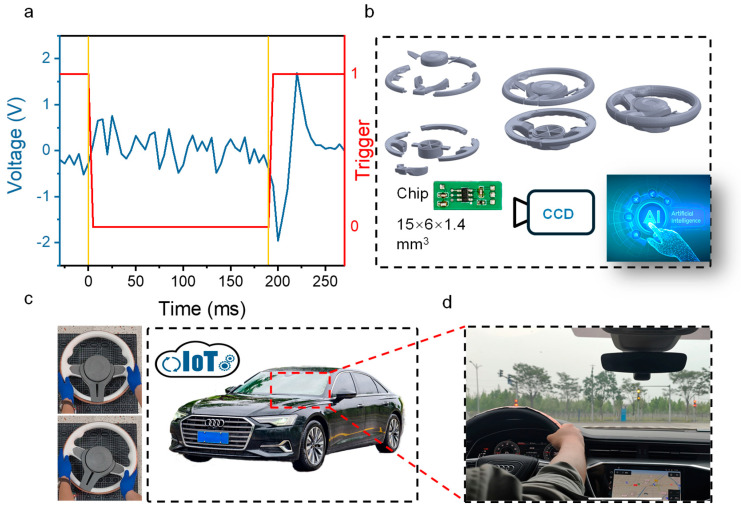
The application of the smart surface in driving scenery. (**a**) The signal diagram of the smart surface. (**b**) The structural decomposition diagram of 3D−printed steering wheel and IoT circuit, and the illustration of monitoring user action by optical image sensor, IoT network and AI algorithm. (**c**) Vehicle and (**d**) on−road demonstration of smart surface.

Figure 4 shows the applications of the smart surface. Figure 4a shows the signal diagram. The red line drops from ‘1’ to ‘0’ at 0, referring to the user raising his hand from the steering wheel. The TENG signal (blue line) exhibits a peak. And, when the time is 190 ms, the red signal rises to ‘1’ when the user touches the steering wheel again, the TENG signal shows the peak again.

Figure 4b shows the 3D-printed steering wheel structure and the IoT chip. To evaluate the performance of the smart surface, a full-scale (1:1) automotive steering wheel model was fabricated using fused deposition modeling (FDM) 3D printing technology (X1C, Bambu Lab) with polylactic acid (PLA) filament. The model design was sourced from open-source community repository (makerworld.com (accessed on 1 September 2025)) with the Profile ID 997121. A TTP223 touch pad detector IC was employed to monitor the voltage signal generated by the TENG. The IC was connected to a Xiaomi IoT switch module. When the driver’s hand touches or releases the steering wheel, the TENG sensor on the surface generates a voltage signal, which is detected and digitized by the TTP223 IC. The IC then triggers the IoT switch, activating the Xiaomi camera for image acquisition.

TENG sensors can be directly embedded within the steering wheel structure during the 3D printing process. The smart surface attached to the steering wheel, the IoT and the CCD capture and analyze the signal to identify whether the driver is holding the steering wheel. When signals are generated by the TENG sensors on the steering wheel due to the driver’s hand detachment or other factors, the IoT circuit is triggered. This activation initiates the CCD to capture optical image information surrounding the steering wheel. Machine learning algorithms subsequently analyze these images to accurately determine whether the signal corresponds to: (a) legitimate driver hand disengagement during autonomous driving (requiring alert activation) or (b) a false positive induced by signal interference or other artifacts (requiring dismissal). The smart surface was tested in actual vehicles, as shown in Figure 4c,d. The photograph of steering wheel holding detection monitoring systems is shown in Figure S3. The detection results of hands-on or hands-off steering wheel are shown in Figure S4.

## 3. Conclusions

In this study, the machine learning and TENG technology are used to create an intelligent surface sensing system. In order to track driver behavior in real time, the system works with an optical image recognition module and uses a self-powered TENG sensor circuit as an event trigger. Compared to continuous video surveillance, the method reduces onboard computational burden, addresses privacy concerns, and detects hazardous behaviors like hands leaving the driving wheel. TENG sensors can work as intelligent triggers instead of passive sensing units, allowing for effective AI synergy. This method not only guarantees precise and dependable driver monitoring, but it also frees up important vehicle computing resources for safety-critical tasks like sophisticated driver assistance systems.

## Data Availability

The original contributions presented in this study are included in the article/Supplementary Material. Further inquiries can be directed to the corresponding authors.

## Supplementary Materials

The following supporting information can be downloaded at: https://www.mdpi.com/article/10.3390/nano15191472/s1, Figure S1: The photographs of the actual device; Figure S2: Electrical measure-ment instrument; Figure S3: Photograph of steering wheel holding detection monitoring systems; Figure S4: Detecting results of hands-on or hands-off the steering wheel.
