# Supplementary material for: Smart Surface of Steering Wheel Based on Triboelectric Nanogenerator and Artificial Intelligence in Driving Monitoring System
Source: Nanomaterials (Basel). 2025 Sep 25;15(19):1472. doi: 10.3390/nano15191472 (PMC12525957; doi:10.3390/nano15191472)
Supplement: Supplementary file 1 [file nanomaterials-15-01472-s001.zip › nanomaterials-3883782-supplementary.pdf]

nanomaterials

MDPI

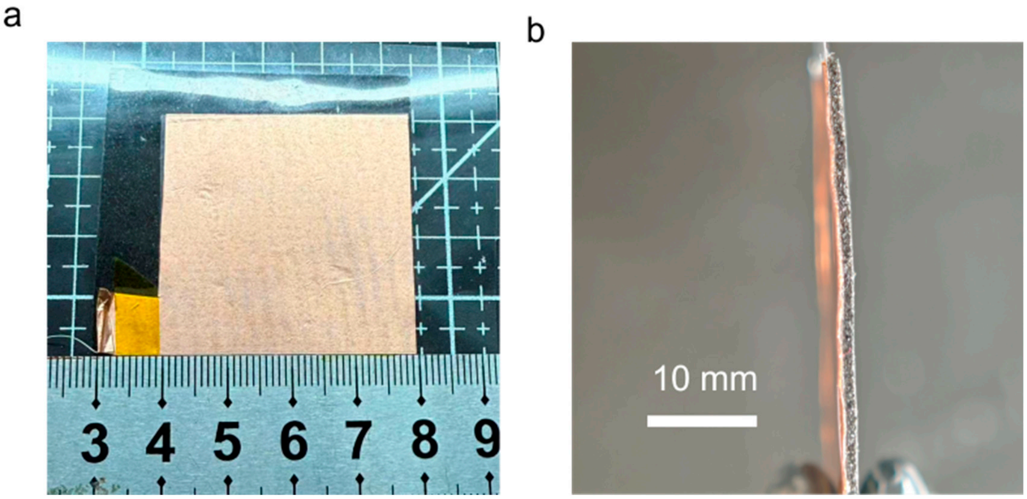

Figure S1. The photographs of the actual device. (a) The front view. (b) The cross-sectional view.

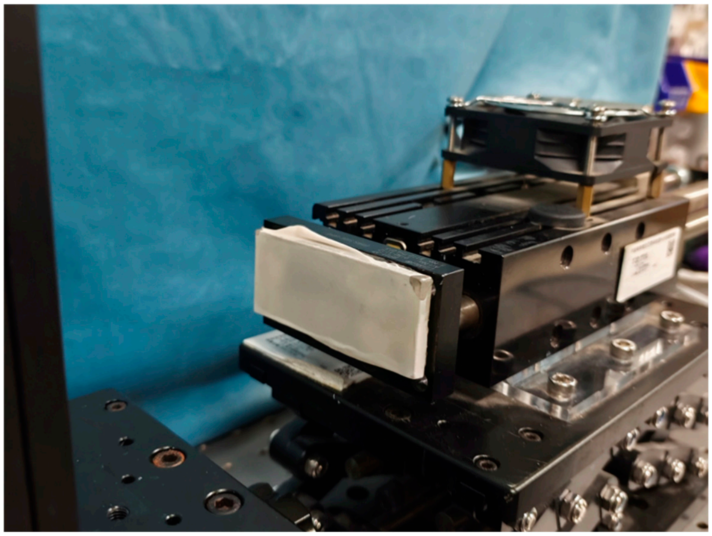

Figure S2. Characterization and electrical measurement instrument.

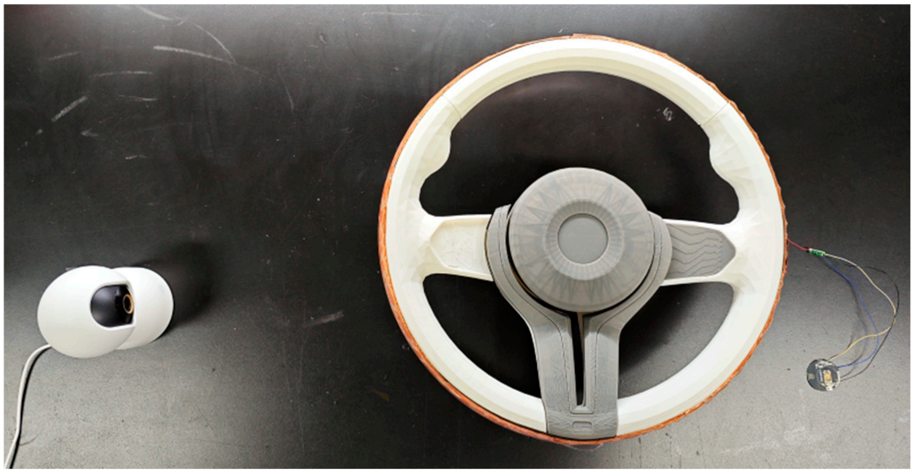

Figure S3. Photograph of steering wheel holding detection monitoring systems.

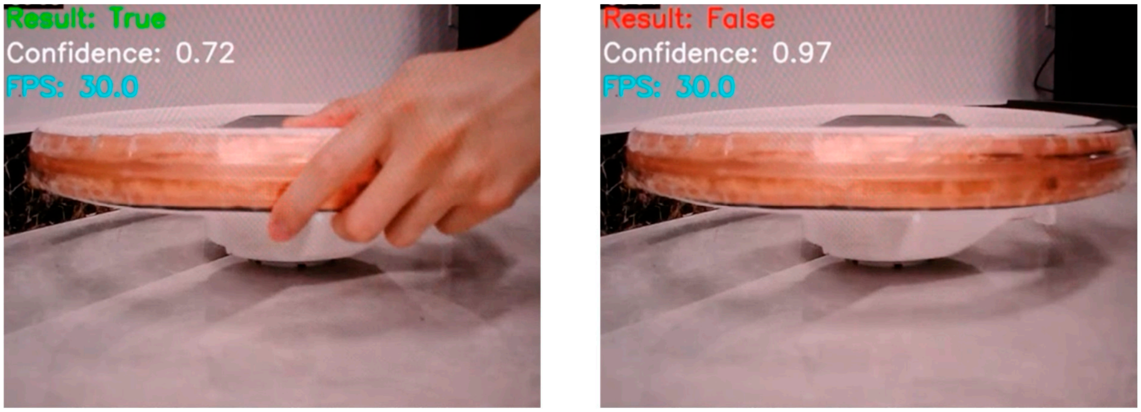

Figure S4. Detecting results of hands-on or hands-off the steering wheel.
